# The joint subclinical elevation of CRP and IL-6 is associated with lower health-related quality of life in comparison with no elevation or elevation of only one of the biomarkers

**DOI:** 10.1007/s11136-015-1068-6

**Published:** 2015-07-21

**Authors:** Peter Garvin, Evalill Nilsson, Jan Ernerudh, Margareta Kristenson

**Affiliations:** Unit of Research and Development in Local Health Care (FoU-enheten för Närsjukvården i Östegötland), County of Östergötland, 581 85 Linköping, Sweden; Division of Community Medicine, Department of Medical and Health Sciences, Linköping University, Linköping, Sweden; Division of Neuro and Inflammatory Sciences, Department of Clinical and Experimental Medicine, Linköping University, Linköping, Sweden

**Keywords:** Biomarkers, CRP, Health-related quality of life, Inflammation, Interleukin, Population, SF-36

## Abstract

**Background:**

Measures of health-related quality of life (HRQoL), like the Short Form (SF)-36, have been suggested to correlate with inflammatory biomarkers. It is, however, unclear whether a joint measure of two inflammatory biomarkers would bring additional information in comparison with evaluation of one inflammatory biomarker.

**Objective:**

To evaluate associations between SF-36 and low-grade inflammation in a Swedish population, with emphasis on a combined measure of C-reactive protein (CRP) and interleukin-6 (IL-6) as a proxy for low-grade inflammation.

**Methods:**

In a randomly selected sample of a middle-aged Swedish general population (*n* = 905; aged 45–69 years, 50 % women), relations between SF-36 parameters and the biomarkers were tested. Regression and correlation analyses were adjusted for sex, age, presence of disease, lifestyle, and psychological factors.

**Results:**

After adjustment for sex and age, HRQoL was significantly lower in the group with a joint elevation of CRP and IL-6 in comparison with either the group with no elevation or the groups showing elevation of one of the two biomarkers. Also after full adjustments, the combined measure of elevated CRP and IL-6, with few exceptions, was associated with significantly lower HRQoL in comparison with elevations in one of them, difference ranging from 4 (Mental Health scale) to 18 scale steps (Role-Physical scale).

**Conclusion:**

This study confirms that there is a relationship between HRQoL and low-grade inflammation. In particular, SF-36 scores are significantly lower in a group with joint elevation of IL-6 and CRP, in comparison with elevation of either one of them.

## Introduction

Measures of health-related quality of life (HRQoL) are increasingly used as patient-reported outcome measures (PROM) within the health-care system, and the assessment constitutes a potentially important tool for health-care improvement. Profound understanding of the relations of HRQoL to other (health-related) factors is therefore crucial for the interpretation of HRQoL data. HRQoL measures can be either generic, measuring general health problems, or disease-specific, i.e., measuring health issues of importance for a certain disease or condition. One of the most commonly used generic measures is the Medical Outcome Study Short Form-36 (SF-36) questionnaire [1], a multi-item measure which includes 36 questions in eight different health dimensions (scales), covering physical, social, and mental function and well-being.

Several groups have independently shown that low SF-36 scores are associated with increased mortality risk [2–4]. We have previously shown that each of the scales of the SF-36, in addition to lifestyle factors and the presence of disease, can be explained to a significant extent by psychological factors, in a similar way for both men and women [5].

Advances within the field of psychoneuroimmunology have led to a detailed knowledge about behavioral–neural–endocrine–immune system interactions [6]. While psychosocial stress and negative emotions are, themselves, predictors for illness and mortality [7], they have also been related to increased levels of inflammatory cytokines, e.g., interleukin (IL)-6 [8, 9]. Interleukin-6 (IL-6) is a pleiotropic cytokine, affecting several biological processes. Besides the induction of C-reactive protein (CRP), an acute-phase protein widely used as a biomarker of inflammation, IL-6 is also linked to the process of inducing “sickness behavior,” a term given to the process whereby the cytokines affect sleep, eating behavior, and mood states, making a person experience feelings of “sickness,” discomfort, and low energy [10], all suggested to be associated with low self-rated health (SRH) [11].

It has been suggested independently by several groups that there is an association between HRQoL and inflammatory biomarkers. This has been shown for SRH [12–15], but also for more elaborated instruments: Both the SF-36, or other generic HRQoL instruments in the same family, as well as various disease-specific HRQoL instruments have been used in studies where it has been postulated that the relation between HRQoL and mortality includes a psychoneuroimmunological pathway. However, although there is some empirical support for the associations between various HRQoL measures and cytokines, results have been inconsistent and inconclusive [16–26]. Similarly, inconsistent results have also been found regarding the association between HRQoL and CRP [26–30]. This may, at least in part, be explained by the heterogeneity of the patient groups studied, as well as rather small study populations.

As far as we know, there is only one large-scale study using a combination of inflammatory biomarkers when evaluating the association between HRQoL and low-grade inflammation.

A population-based study of the relation between fatigue [measured using the vitality scale (VT) of the SF-36 questionnaire] and a joint elevation of CRP and IL-6 showed a greater risk of a low score of the VT scale, than for elevated levels of CRP or IL-6 separately. The other scales of the SF-36 were not evaluated in this study [31].

In parallel, low-grade inflammation has been found to predict mortality in older people, independently of the presence of disease [32, 33]. Of note, the combination of elevated levels of both IL-6 and CRP has been found to be associated with a higher mortality risk than measures based on either of the biomarkers alone [33].

The aim of the present study was to evaluate associations between HRQoL and low-grade inflammation in a Swedish general population. In particular, we aimed to investigate a combined measure of CRP and IL-6 in comparison with one biomarker at a time. Our hypothesis was that a combination of CRP and IL-6 would have stronger associations with low HRQoL, than evaluating each biomarker separately.

## Methods

### Subjects

Data were collected between October 2003 and May 2004, using random sampling (stratified according to the catchment areas of 10 different primary health-care centers, sex, and age at 5-year intervals) of the population in the county of Östergötland, Sweden. An invitation letter was sent by post, while signing and returning a reply form provided informed consent. Participants were enrolled until the predetermined sample size of 500 women and 500 men between the ages 45 and 69 was obtained (five 5-year age groups with 50 women and 50 men in each), and finally resulted in 505 women and 502 men. This constituted the basis of the ongoing, prospective Life conditions, Stress, and Health (LSH) study, with a response rate of 62.5 % [34]. The LSH study was designed to investigate whether the relationship between socioeconomic status and coronary heart disease might be mediated through biopsychosocial pathways. Since the primary outcome in this study is coronary heart disease, the age group 45–69 was chosen to optimize the number of outcomes. Exclusion criteria were severe physical or mental disease, or difficulties in understanding the Swedish language. The study sample was representative of the population in terms of educational attainment, immigrant status, and employment rates.

### Procedures

As part of the protocol of the LSH study, participants visited their primary health-care centers, where anthropometric and blood pressure measurements, in addition to blood, urine, and saliva samples, were obtained. All samples were collected in a fasting state. Out of the 1007 participants, blood samples were eligible for analysis from 961 participants.

Patients were instructed to reschedule their appointment if they were currently experiencing any acute infections, e.g., a common cold. At the visit, information about the voluntariness of participating in the study was given verbally. In order to ensure standardization of the data collection, nurses collecting data at the 10 primary health-care centers were trained together.

Except for sex and age, all other measures in the present study were self-reported (from questionnaires).

### Measures

#### HRQoL

The Swedish standard version 1 of SF-36 [1, 35] was used to measure HRQoL. Items are aggregated into eight multi-item scales, called Physical Functioning (PF), Role-Physical (RP), Bodily Pain (BP), General Health (GH), Vitality (VT), Social Functioning (SF), Role-Emotional (RE), and Mental Health (MH). Items were transformed and summed according to the manual of the instrument, to give scores from 0 to 100 for each scale, with a higher score indicating a better HRQoL [1, 35].

#### Biological markers

Plasma levels (EDTA plasma) of IL-6 were measured with ultrasensitive bead kit technology (Invitrogen Co, Carlsbad, CA, USA) according to the manufacturer’s instructions and analyzed on a Luminex^®^ 100™ system (Austin, TX, USA). The lower limit of detection was 1.68 pg/ml and inter-assay coefficient of variance (CV) of 7.0 %, and proportion of samples with detectable levels was 40 %. Samples below the detection levels were given a value that was a third of the detection limit.

C-reactive protein (CRP) was measured in EDTA plasma by a highly sensitive latex-enhanced turbidimetric immunoassay (Roche Diagnostics GmbH, Vienna, Austria) with a lower detection limit of 0.03 mg/l and CV of 1.7 %. Detectable levels were found for all samples but one, which received a value of zero.

Aliquots of plasma (0.5 ml) were stored in −70° Celsius approximately 40 months before analysis.

#### Presence of disease

The concept “Presence of disease” included two different variables. The first variable, “Medical conditions,” was defined as referring to a participant having at least one of 14 medical conditions. Data in the LSH study on medical conditions were self-reported using a checklist. Participants were asked whether they had ever been diagnosed by a physician with any of the 12 following medical conditions: myocardial infarction, angina pectoris, stroke, chronic obstructive pulmonary disease, cancer, asthma/allergy, dyspepsia/peptic ulcer, kidney disease, celiac disease, hypertension, hyperlipidemia or diabetes mellitus. An open question asking about the presence of other medical conditions than the above concluded the checklist. Rheumatic disease and fibromyalgia were common enough to be added to our variable “Medical conditions.” Another aspect of illness important to HRQoL is pain, and the questionnaire contained a question about the presence of musculoskeletal pain in the back of the neck and/or in the back (henceforth referred to only as back pain), constituting the second variable, “Back pain.”

#### Lifestyle factors

Smoking habits were categorized as being either a smoker (at least one cigarette a day) or not. Those who had quit smoking within the last 5 years due to illness were also included in the smoker category. Hazardous alcohol consumption was defined as drinking ≥9/14 (women/men) standard glasses/week (one standard glass = 12 g of alcohol). Those who had abstained from alcohol due to illness were also included in this category. Questions about alcohol consumption were part of a validated Food Frequency Questionnaire [36]. Physical activity was measured by questions about daily physical activity (four levels) and planned physical exercise (five levels). Responses were then combined into four activity levels: regularly active, occasionally active, seldom active or inactive [37]. For the present study, occasionally and seldom active were then merged into one category. Body mass index (BMI) was used as a measure of weight control and categorized as <25, 25–30, or >30. Weight and height used for calculating BMI were measured by the nurses during visits to the primary health-care centers.

#### Psychological factors

The questionnaires in the LSH study included a broad range of instruments measuring psychosocial variables. Earlier [5] we found that, after adjustment for effects of age, sex, presence of disease, and lifestyle, the following two instruments (dimensions) were among those that contributed most strongly to the variation of SF 36 scores, and therefore they were included in the present study. One instrument represents psychological risk factors (risk factors for disease; lower scores being favorable). This is *The Center for Epidemiologic Studies Depression scale* (CES-D) [38], which was developed in the 1970s to capture depressed moods in epidemiological studies. The other instrument measures resources (that supposedly protect against disease; higher scores being favorable). This is *Sense of Coherence* (SoC), which reflects the extent to which one feels one’s own life to be comprehensible, manageable, and meaningful [39].

### Statistical methods

Prior to analyses, cutoffs were set at >10 mg/l for CRP and >20 pg/ml for IL-6, to exclude participants with an acute inflammatory response due to, e.g., ongoing infections (*n* = 32 for CRP, *n* = 17 for IL-6).

Descriptive data include mean (SD; range) and frequency.

Partial correlation analyses, adjusted for age and sex, were used to explore the relation between the SF-36 and the biomarkers, and a Fisher *r*–*z* transformation was used to investigate differences in correlation coefficients between women and men.

The data set was considered to be large enough to use linear regression analyses on SF-36 scores [40]. Linear regressions (adjusted for age and sex) were used to explore the relation between the SF-36 and the explanatory variables used in the regression analyses, i.e., inflammatory biomarkers presence of disease, lifestyle, and psychological factors. The two biomarkers were evaluated one at a time. The combined effect of CRP and IL-6 was examined by creating a composite variable with four categories: low CRP and low IL-6, high CRP and low IL-6, low CRP and high IL-6, and high CRP and high IL-6. Low CRP was defined as CRP <1 mg/l, and high CRP as >3 mg/l, using the Centers for Disease Control/American Heart Association (CDC/AHA) criteria regarding risk assessments for cardiovascular disease [40]. To define high IL-6 levels in the present study, we used the same proportion of study participants as found for the high category of CRP. This corresponds to an IL-6 level of 3.25 pg/ml. As the levels to define high and low levels of the biomarkers are arbitrary, four altered definitions were used to categorize an elevation of the biomarkers, using (a) highest quartile (CRP > 2.26 mg/l and IL-6 > 2.58 pg/ml), (b) median split (CRP > 0.85 mg/l and IL-6 > 0.56 pg/ml), (c) the same absolute cutoffs as used in the article by Cho et al. (CRP > 1.0 mg/l and IL-6 > 1.5 pg/ml) [31], and (d) the same absolute cutoffs as used in the article by Harris et al. [33] (CRP > 2.78 mg/l and IL-6 > 3.15 pg/ml).

A two-sided probability value of *p* ≤ 0.05 was considered as statistically significant. Analyses were performed in STATA statistical software, release 11.0 (Stata Corporation, College Station, TX, USA), IBM SPSS for Windows statistical software, release 21 (IBM Corporation, Armonk, NY, USA).

## Results

Descriptive data regarding the study sample (*n* = 905) are given in Table 1. Significant sex differences were seen for many of the variables in Table 1 (back pain, BMI, alcohol consumption, CRP, and all scales of the SF-36 except the RE scale). Therefore, correlation analyses between the SF-36 and the biomarkers were first carried out separately for women and men. However, the Fischer *r*–*z* transformation did not show any significant differences in the correlation coefficients for women and men, and separating the analyses by sex did not change the main findings. Hence, further analyses were adjusted for sex, but were not performed separately for women and men.

**Table 1:** Characteristics of the study population (*n* = 905)

	%	*n*
Sex		
Women	50	454
Age		
45–49	20	179
50–54	21	188
55–59	20	185
60–64	20	178
65–69	19	175
Medical conditions		
One or more of all diseases	55	497
Back pain		
Yes	41	375
Smoking		
Yes	21	193
Alcohol consumption		
9 (women)/14 (men) or more glasses/week	10	86
Body mass index		
<25	38	340
25–30	43	385
>30	19	171
Physical activity		
Inactive	4	36
Occasionally/seldom active	76	640
Regularly active	19	163

	Mean	SD	Range
Psychological instruments			
CES-D, Center for Epidemiologic Studies Depression scale (possible range 0–60)	8.8	7.7	0–51
SOC, Sense of Coherence (possible range 13–91)	68.8	10	32–91
SF-36			
PF, Physical Functioning (possible range 0–100)	84.7	18	0–100
RP, Role-Physical (possible range 0–100)	81.6	34	0–100
BP, Bodily Pain (possible range 0–100)	69.2	26	0–100
GH, General Health (possible range 0–100)	70.5	21	5–100
VT, Vitality (possible range 0–100)	66.6	23	0–100
SF, Social Functioning (possible range 0–100)	87.8	20	0–100
RE, Role-Emotional (possible range 0–100)	86.2	29	0–100
MH, Mental Health (possible range 0–100)	79.3	17	8–100
Biomarkers			
IL-6 (pg/ml)	1.9	2.5	0.56–19.5
CRP (mg/l)	1.7	2.0	0–9.9

	%	*n*
Combinations of CRP and IL-6 (*n* = 534)		
Low CRP low IL-6 (CRP < 1 mg/l, IL-6 < 0.57 pg/ml)	67	355
High CRP low IL-6 (CRP > 3 mg/l, IL-6 < 0.57 pg/ml)	12	66
Low CRP high IL-6 (CRP < 1 mg/l, IL-6 > 3.25 pg/ml)	10	52
High CRP high IL-6 (CRP > 3 mg/l, IL-6 > 3.25 pg/ml)	11	61

The results of partial correlations (adjusted for age and sex) between the scales of the SF-36 and the biomarkers are shown in Table 2. Significant correlation coefficients for every scale of the SF-36 were found for IL-6, whereas CRP showed significant relations for all scales except the MH scale.

**Table 2:** Partial correlation between the SF-36 and the inflammatory biomarkers, adjusted for age and sex

SF-36	IL-6		CRP	
	*r*	*p*	*r*	*p*
PF, Physical Functioning	−**0.18**	<**0.001**	−**0.15**	<**0.001**
RP, Role-Physical	−**0.09**	**0.005**	−**0.12**	<**0.001**
BP, Bodily Pain	−**0.10**	**0.001**	−**0.12**	<**0.001**
GH, General Health	−**0.15**	<**0.001**	−**0.12**	<**0.001**
VT, Vitality	−**0.10**	**0.001**	−**0.07**	**0.023**
SF, Social Functioning	−**0.13**	<**0.001**	−**0.09**	**0.005**
RE, Role-Emotional	−**0.13**	<**0.001**	−**0.08**	**0.009**
MH, Mental Health	−**0.12**	<**0.001**	−0.02	0.393

Statistically significant correlations are shown in bold. *n* = 855–905

All lifestyle and psychological factors, as well as presence of disease, were related to HRQoL, as expected (Table 3). High alcohol consumption had the lowest coefficients in regard to SF-36, but still significant to five of the eight scales.

**Table 3:** SF-36 in relation to potential explanatory variables

Explanatory factors		SF-36							
		PF	RP	BP	GH	VT	SF	RE	MH
Medical conditions (y/*n*)		−**6.5**	−**12.5**	−**8.8**	−**11.1**	−**8.7**	−**6.2**	−**5.4**	−**3.6**
	*p*	<**0.001**	<**0.001**	<**0.001**	<**0.001**	<**0.001**	<**0.001**	**0.008**	**0.002**
Back pain (y/*n*)		−**10.7**	−**17.8**	−**27.1**	−**14.4**	−**14.7**	−**7.1**	−**6.9**	−**5.4**
	*p*	**0.001**	**0.001**	**0.001**	**0.001**	**0.001**	**0.001**	**0.001**	**0.001**
Smoking (y/*n*)		−**7.3**	−**8.8**	−**8.2**	−**6.3**	−**7.2**	−**5.8**	−**9.3**	−**6.1**
	*p*	**0.001**	**0.001**	**0.001**	**0.001**	**0.001**	**0.001**	**0.001**	**0.001**
High alcohol consumption (y/*n*)		−**5.4**	−**9.5**	−3.1	−**5.1**	−4.6	−**6.9**	−**11.2**	−3.6
	*p*	**0.008**	**0.015**	0.304	**0.035**	0.076	**0.002**	**0.001**	0.069
BMI (three categories)		−**5.8**	−**7.0**	−**5.4**	−**5.8**	−**4.3**	−**3.0**	−**3.2**	−1.1
	*p*	**0.001**	**0.001**	**0.001**	**0.001**	**0.001**	**0.001**	**0.020**	0.144
Physical activity (three categories)		**11.2**	**9.7**	**7.6**	**10.0**	**8.8**	**4.2**	**4.8**	**2.6**
	*p*	**0.001**	**0.001**	**0.001**	**0.001**	**0.001**	**0.002**	**0.032**	**0.042**
Sense of coherence		**3.5**	**7.7**	**4.6**	**8.2**	**9.5**	**6.8**	**10.9**	**8.9**
	*p*	**0.001**	**0.001**	**0.001**	**0.001**	**0.001**	**0.001**	**0.001**	**0.001**
CES-D		−**4.7**	−**10.9**	−**6.3**	−**10.0**	−**13.2**	−**10.5**	−**16.5**	−**12.4**
	*p*	**0.001**	**0.001**	**0.001**	**0.001**	**0.001**	**0.001**	**0.001**	**0.001**

Regressions adjusted for age and sex. Beta coefficients expressed as change per category (dichotomy, or three categories for body mass index and physical activity) or per SD increment in continuous scales (psychological factors). Statistically significant findings are shown in bold. *n* = 878–976

SF-36: *PF* Physical Functioning, *RP* Role-Physical, *BP* Bodily Pain, *GH* General Health, *VT* Vitality, *SF* Social Functioning, *RE* Role-Emotional, *MH* Mental Health, *CES-D* Center for Epidemiologic Studies Depression scale

**Fig. 1:**
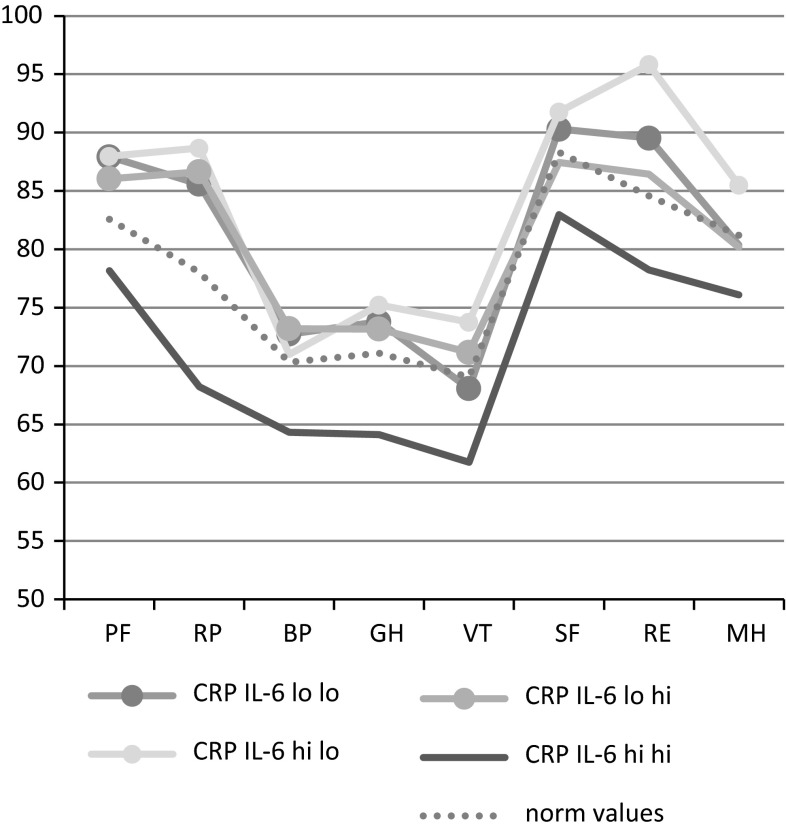
Mean SF-36 scale scores for different combinations of CRP and IL-6, adjusted for age, sex, presence of disease, lifestyle factors, and psychological factors. *n* = 435–457. SF-36: *PF* Physical Functioning, *RP* Role-Physical, *BP* Bodily Pain, *GH* General Health, *VT* Vitality, *SF* Social Functioning, *RE* Role-Emotional, *MH* Mental Health. *CRP* C-reactive protein, *IL* interleukin. CRP hi = 3–10 mg/l, CRP lo ≤ 1 mg/l, IL-6 hi = 3.25–20 pg/ml, IL-6 lo ≤ 0.56 pg/ml. *Dashed line* represents Swedish norm values standardized to the gender and age distribution in the study population. Significant differences in group comparisons in relation to group with elevated levels of both CRP and IL-6 are marked with a *filled circle*. The group with elevated levels of both CRP and IL-6 has significantly lower mean levels of SF-36 scores than other groups in 19 out of 24 comparisons (all except BP for CRP IL6 hi lo, SF, RE and MH for CRP IL6 lo hi, and MH for CRP IL-6 lo lo)

In Table 4, the combined measure of CRP and IL-6 in relation to SF-36 scale scores is shown. When running dummy variables of the combined variable adjusted for age and sex, the group with both elevated CRP and elevated IL-6 has significantly lower SF-score for all the subscales than any other group. After full adjustments, scale scores are consistently significantly lower in 19 out of 24 comparisons for the group with both elevated CRP and IL-6. In Fig. 1, the mean scores are shown for each group, along with age-standardized norm values [41]. The groups with either no elevation or with elevation of one of the biomarkers have adjusted mean values that are higher or in the same range as the norm values, whereas the group with both elevated CRP and elevated IL-6 is considerably lower.

**Table 4:** SF-36 for different combinations of CRP and IL-6

SF-36		High CRP low IL-6 (*n* = 66)		Low CRP high IL-6 (*n* = 61)		Low CRP low IL-6 (*n* = 355)		*R* ^2^ values	
		Model a	Model b	Model a	Model b	Model a	Model b	Model a	Model b
PF		**10.7**	**9.8**	**10.7**	**8.1**	**14.7**	**9.7**	***0.12***	***0.33***
	*p*	<**0.001**	<**0.001**	<**0.001**	**0.005**	<**0.001**	<**0.001**		
RP		**14.8**	**17.1**	**20.7**	**18.1**	**20.0**	**17.1**	***0.04***	***0.20***
	*p*	**0.015**	**0.006**	**0.001**	**0.004**	<**0.001**	**0.001**		
BP		**10.3**	5.4	**16.2**	**8.8**	**17.0**	**7.9**	***0.06***	***0.38***
	*p*	**0.030**	0.200	**0.001**	**0.040**	<**0.001**	**0.028**		
GH		**12.1**	**11.0**	**15.1**	**9.4**	**16.1**	**9.7**	***0.07***	***0.38***
	*p*	**0.002**	**0.002**	<**0.001**	**0.008**	<**0.001**	**0.001**		
VT		**9.1**	**11.3**	**12.6**	**9.9**	**11.0**	**6.1**	***0.09***	***0.47***
	*p*	**0.026**	**0.001**	**0.002**	**0.005**	**0.001**	**0.035**		
SF		**8.0**	**8.1**	**8.5**	4.2	**11.9**	**7.0**	***0.07***	***0.35***
	*p*	**0.024**	**0.013**	**0.018**	0.206	<**0.001**	**0.010**		
RE		**13.9**	**16.7**	**11.3**	8.0	**15.4**	**11.2**	***0.03***	***0.26***
	*p*	**0.011**	**0.002**	**0.040**	0.145	**0.001**	**0.014**		
MH		**8.8**	**8.9**	**7.1**	3.9	**8.1**	4.2	***0.07***	***0.49***
	*p*	**0.007**	**0.001**	**0.031**	0.152	**0.002**	0.062		

Beta coefficients represent differences in SF-36 score for dummy variables in comparison with the group with high CRP and high IL-6 (*n* = 52). Model a adjusted for age and sex. Model b adjusted for age, sex, presence of disease, lifestyle factors, and psychological factors. Statistically significant findings are shown in bold

R2, coefficient of determination, is shown for model a and b for each of the scales in SF-36 are in bold italic

The smallest differences were seen for the Mental Health scale (where only one of the three comparisons was significant), and the most pronounced difference was found for physical role, where the difference in scale scores to the other groups was about −17 to −18 scale steps.

Four altered ways to define high vs low levels were used to out-rule potential spurious findings due to arbitrary cutoffs. Results were largely unchanged for two of them, namely using either the highest quartile (CRP > 2.26 mg/l and IL-6 > 2.58 pg/ml) or the same absolute cutoffs as used in the article by Harris et al. [33] (CRP > 2.78 mg/l and IL-6 > 3.15 pg/ml). These two approaches for altered cutoffs had significantly lower SF-36 scores in the group with elevated levels in both biomarkers, with bodily pain as only exception (data not shown).

However, for median split (CRP > 0.85 mg/l and IL-6 > 0.56 pg/ml) and the same absolute cutoffs as used in the article by Cho et al. [31] (CRP > 1.0 mg/l and IL-6 > 1.5 pg/ml), there were significant differences only for social function and emotional role function, with nonsignificant differences in the other comparisons (data not shown).

Adjusting for physical activity and BMI either as ordinal categories or dummy variables had no major impact on any of the above regressions.

## Discussion

In the present study, we found significant negative correlations (after adjustment for age and sex) for all scales of the SF-36 to plasma levels of IL-6 and CRP (except the MH scale for CRP). The combined measure of both elevated CRP and IL-6 was associated with markedly lower SF-scores than the groups with elevation of either one of them. The significantly lower SF-scores for the group with subclinical elevations of both CRP and IL-6 remained after adjustments for medical conditions, lifestyle factors, and psychological factors.

Our findings corroborate the few earlier population-based studies using a combined measure of inflammatory biomarkers rather than looking at biomarkers one by one.

Harris et al. [33] found that in a population of older nondisabled persons (age 77 ± 3.2 years), there was a 2.6 times greater mortality for the joint elevation of high but subclinical CRP ((fourth quartile, ≥2.78 mg/l) and IL-6 (fourth quartile, >3.19 pg/ml) than for lower than median values (<1.57 mg/l and 2.08 pg/ml, respectively). Persons with only high IL-6 also had a higher mortality risk, while persons with only high CRP did not.

In the Whitehall II study, Cho et al. [31] primarily used the same approach as we did to find cutoffs for CRP, namely the CDC/AHA criteria [41]. However, when categorizing the joint elevation, they used the cutoffs high CRP ≥ 1 mg/l and high IL-6 ≥ 1.5 pg/ml. Fatigue was defined as scoring ≤50 on the VT scale of the SF-36 (28 % of the study population). Of the respondents with no fatigue at baseline, 20 % scored ≤50 at follow-up. Persons with both high CRP and high IL-6 had significantly higher risk of developing fatigue than persons with only low values, while those with only one elevated biomarker did not. Thus, similar to our study, the joint elevation of CRP and IL-6 had a stronger association with VT than was the case for elevation in just one of the biomarkers.

Both Harris et al. [33] and Cho et al. [31] used well-characterized study populations, allowing them to adjust for a number of relevant factors, similar to our approach. In all three studies, the relations remained after the adjustments, implying that there seems to be a biological link between low-grade inflammation and HRQoL. In particular, although the studies use in part different methods and biomarker levels, both Harris et al. [33] and Cho et al. [31] indicate that neither CRP nor IL-6 alone is a sufficient measure and that a combined measure of the two is superior. Our data confirm that subclinical elevation in only one of these two biomarkers may be cumbersome to evaluate as there are small differences in comparison with the group without any elevation, whereas an elevation in both may be a better marker for sustained elevations in low-grade inflammation.

There were no major differences between the cutoffs used based on proportion corresponding to CRP > 3 mg/l, highest quartile or categorization used by Harris [33]. This is to be expected, as all these three arbitrary cutoffs are close to one another in terms of absolute comparisons. In contrast, our findings were altered substantially when using median split or the categorization used by Cho [31]. This is also to be expected, as both these categorizations include a high number of participants as “high” although inflammation is relatively low (starting from CRP > 1 mg/l). We propose that the proportion having 3 mg/l or higher for CRP could be used to set the cutoff for IL-6. An absolute level of IL-6 may be cumbersome to define, due to difficulties in measurements of low concentrations and variations across methodology used.

It might be argued that the large effect of combining the two biomarkers is unexpected, since IL-6 is one of the main inducers to release CRP. Therefore, elevations of CRP reflect elevations of IL-6 to a substantial extent, and the correlation between the two should be high. However, importantly, the kinetics differ between the two biomarkers, both in release patterns as well as concentrations in circulation, where both fluctuates considerably but IL-6 has a much lower half-life than CRP. In samples from a normal population, we might speculate that elevations in one of the two biomarkers to a large extent reflect functional acute-phase response among certain individuals, whereas elevations in both may be a more valid measure for a long-standing subclinical inflammation. Thus, it would be expected that a combination of the two biomarkers has an impact on the association with HRQoL, whereas a rise in just one of them does not.

A potential limitation of the study was that the proportion of samples with detectable plasma levels for IL-6 was low, which might negatively influence the correlation and regression analyses. However, IL-6 still showed consistent and highly significant correlations to SF-36. Unarguably, difficulties with the measurement bring limitations to explore appropriate cutoff levels for IL-6. However, this limitation does not have an impact on our major finding, that the combination of the two biomarkers has a stronger association with HRQoL, than the elevation of either one of them.

## Conclusion

This study demonstrates that there is a relationship between HRQoL and low-grade inflammation. In particular, the association is significantly stronger when using a combined measure of IL-6 and CRP in comparison with elevation of either one of them.
